# Seafood-Linked and Sex-Specific Signatures of Legacy and Emerging PFAS Body Burden During Dietary Transition in Sichuan, China

**DOI:** 10.3390/foods15142472

**Published:** 2026-07-13

**Authors:** Run Li, Zhan Lu, Sisi Wang, Jing-Guang Li, Sheng Wen, Shen-Sheng Xiao, Lei Zhang, Xin Liu, Yong-Ning Wu

**Affiliations:** 1 College of Food Science and Engineering, Wuhan Polytechnic University, Wuhan 430023, China; 13260917031@163.com (R.L.); zhanlu0409@gmail.com (Z.L.); wangsisi692@gmail.com (S.W.); wuyongning@cfsa.net.cn (Y.-N.W.); 2 NHC Key Lab of Food Safety Risk Assessment, China National Center for Food Safety Risk Assessment, Beijing 100010, China; lijg@cfsa.net.cn (J.-G.L.); zhanglei1@cfsa.net.cn (L.Z.); 3 NHC Specialty Laboratory of Food Safety Risk Assessment and Standard Development, Hubei Provincial Center for Disease Control and Prevention, Wuhan 430079, China; wenshenggy@aliyun.com

**Keywords:** PFAS, seafood consumption, dietary transition, human biomonitoring, emerging contaminants, food safety

## Abstract

Per- and polyfluoroalkyl substances (PFASs) are persistent contaminants of concern in food safety and human exposure assessment. Aquatic foods are important components of dietary diversification, but their association with internal PFAS burden remains unclear in inland populations. Of the 1294 participants initially recruited, 1292 with complete demographic information were included in the final analyses. Serum concentrations of 22 PFASs were measured by solid-phase extraction coupled with UPLC-MS/MS, and nine PFASs detected in more than 75% of samples were analyzed. Dietary intake frequency, sociodemographic characteristics, and lifestyle factors were collected using structured questionnaires. Multivariable linear regression and sex-stratified analyses were performed. Seafood-related food groups showed the most consistent positive associations with serum PFAS levels. Frequent consumption of shellfish, shrimp, crabs, fish, and seaweed was associated with higher concentrations of long-chain PFASs, including PFDA, PFNA, PFHxS, PFOS, and PFOA, while shrimp and crab intake was also positively associated with 6:2 Cl-PFESA. These associations were generally stronger in males, suggesting sex-related heterogeneity in the relationship between seafood intake and PFAS body burden. These findings suggest that seafood consumption is associated with distinct PFAS body-burden profiles in inland adults and may support integrated biomonitoring, dietary assessment, and food-contaminant surveillance.

## 1. Introduction

Per- and polyfluoroalkyl substances (PFASs) have become a persistent challenge at the interface of environmental contamination, food safety, and human exposure assessment [1]. Owing to their resistance to water, oil, and heat, PFASs have been widely used in industrial applications and consumer products, including non-stick cookware, food-contact materials, textiles, and firefighting foams [2,3]. Their strong carbon-fluorine bonds confer exceptional environmental stability, allowing these compounds to persist in water, soil, air, and biota. Unlike many classical lipophilic persistent organic pollutants, PFAS tend to bind to serum proteins and accumulate mainly in blood, liver, and kidneys [4,5]. Human exposure is therefore not only widespread but also biologically relevant. Epidemiological and toxicological studies have associated PFAS exposure with endocrine disrupting effects [6], reproductive and developmental toxicity [7], neurotoxicity [8], immunotoxicity, and increased cancer risk [9].

Food is considered an important non-occupational factor associated with PFAS body burden in the general population [10,11]. Among different food categories, aquatic foods have received particular attention because PFAS can be transported, bioaccumulated, and biomagnified through aquatic food webs [12]. Fish, shellfish, crustaceans, and seaweed are increasingly consumed as sources of high-quality protein, essential fatty acids, minerals, and other beneficial nutrients. However, these foods may also carry long-chain PFASs and emerging PFAS substitutes, creating a food-safety concern that must be considered alongside their nutritional value. Population-based studies, including analyses from the National Health and Nutrition Examination Survey, have reported positive associations between seafood intake and serum concentrations of perfluorooctane sulfonate (PFOS), perfluorooctanoic acid (PFOA), perfluorononanoic acid (PFNA), and perfluorohexanesulfonic acid (PFHxS) [13]. PFAS have also been detected in vegetables grown in contaminated soils, and experimental evidence supports their uptake by edible plant tissues [14]. Additional dietary factors, including tea, processed meat, and packaged foods, have also been reported to be associated with PFAS concentrations [15]. These findings suggest that PFAS body-burden patterns may be related to modern food systems, dietary diversification, and food supply chains. This issue is particularly relevant in China, where a rapid dietary transition has changed the structure of food consumption [16]. Aquatic foods are no longer consumed mainly in coastal areas. With the development of cold-chain logistics, e-commerce, and national food distribution networks, seafood and other aquatic products have become increasingly accessible to inland populations. Sichuan Province represents a useful setting to examine this transition. Although geographically inland, its residents now have access to and consume more diversified food sources, including fish, shellfish, shrimp, crabs, and seaweed. This shift raises an important question for food safety: whether aquatic food consumption is associated with distinct PFAS body burden profiles in inland populations undergoing dietary transition.

Sex-specific differences add another layer of complexity to the relationship between diet and PFAS body burden. Men and women may differ in food preferences, portion sizes, alcohol and smoking behaviors, occupational activities, body composition, and physiological elimination pathways. Menstruation, pregnancy, and breastfeeding may influence PFAS elimination in women, whereas men may have slower clearance and higher body burdens for some PFAS compounds. Therefore, examining sex-stratified associations between dietary intake and serum PFAS concentrations can help identify heterogeneity in PFAS body-burden profiles and support more targeted risk communication.

In the present cross-sectional study, serum concentrations of nine frequently detected PFAS were measured in 1292 adults from Sichuan Province, China. The primary objective was to evaluate whether aquatic-food-related dietary patterns, including fish, shellfish, shrimp, crabs, and seaweed, were associated with serum PFAS concentrations in this inland population. The secondary objectives were to examine whether these associations differed by sex and to assess whether the emerging PFAS substitute 6:2 Chlorinated Polyfluoroalkyl Ether Sulfonates (6:2 Cl-PFESA) showed dietary association patterns similar to or distinct from those of legacy PFAS. By addressing these objectives, this study aims to provide evidence that may help inform the integration of human biomonitoring with dietary assessment and food-contaminant surveillance, as well as risk-benefit considerations for aquatic foods during dietary transition.

## 2. Materials and Methods

### 2.1. Study Population and Sample Collection

This cross-sectional study included 1292 adult participants recruited from Sichuan Provincial People’s Hospital, China, between August 2020 and June 2021. The study population consisted of 493 males and 799 females. All participants provided written informed consent before enrollment and completed a structured questionnaire covering sociodemographic characteristics, lifestyle factors, and dietary intake frequency.

Fasting venous blood samples (5 mL) were collected by trained medical personnel using standardized procedures. Serum was separated by centrifugation and stored at −80 °C until PFAS analysis. The study protocol was approved by the Ethics Committee of the China National Center for Food Safety Risk Assessment (No. 2019008) and the Ethics Committee of Sichuan Academy of Medical Sciences & Sichuan Provincial People’s Hospital (No. 2020467). Human biospecimen collection was additionally approved by the Ministry of Science and Technology of China in 2020.

### 2.2. Questionnaire Data and Dietary Assessment

Information on sociodemographic characteristics, lifestyle behaviors, and dietary habits was collected using structured questionnaires. Sociodemographic variables included sex, age, body mass index (BMI), education level, annual household income, marital status, and fertility status. Fertility status was assessed based on the self-reported number of children and categorized as no children, one child, two children, or more than two children. Detailed reproductive information, such as pregnancy history, breastfeeding history, breastfeeding duration, age at first birth, or menopausal status, was not collected. Lifestyle variables included smoking, alcohol consumption, physical activity, and sleep quality.

Dietary intake frequency was assessed for 12 common food categories. Particular attention was given to seafood-related foods, including fish, shellfish, shrimp/crabs, and seaweed, because aquatic foods were the major food groups of interest in relation to PFAS exposure. The questionnaire collected intake frequency for these broad aquatic-food categories but did not collect detailed information on specific seafood species, geographic origin or production source, portion size, or cooking methods. Therefore, these variables should be interpreted as frequency-based dietary indicators rather than detailed quantitative measures of seafood-derived PFAS exposure. This limitation is further addressed in the Discussion section. Other food categories included smoked meat, cruciferous vegetables, tea, and vitamin supplements. Intake frequency was categorized into four levels: never, less than once per week, 1–2 times per week, and at least three times per week. For regression analyses, dietary intake variables were dichotomized as high-frequency consumption (≥1 time per week) and low-frequency consumption (<1 time per week). This cut-off was selected to distinguish regular intake from infrequent or non-regular intake and to improve model stability, because some higher-frequency categories contained relatively small numbers of participants for several food groups. The original four-level frequency information was reviewed during data processing, but the binary classification was used as the primary regression approach to facilitate consistent interpretation across multiple dietary variables and PFAS compounds. Because PFAS concentrations were not measured in the consumed food items, these dietary variables were interpreted as intake-related markers associated with serum PFAS concentrations rather than direct indicators of PFAS sources.

### 2.3. PFAS Quantification in Serum

Serum concentrations of 22 PFAS were initially measured. Nine PFAS with detection rates above 75% were selected for statistical analysis: perfluorobutanoic acid (PFBA), perfluorohexanoic acid (PFHxA), perfluorooctanoic acid (PFOA), perfluorononanoic acid (PFNA), perfluorodecanoic acid (PFDA), perfluorobutane sulfonate (PFBS), perfluorohexanesulfonic acid (PFHxS), perfluorooctane sulfonate (PFOS), and 6:2 chlorinated polyfluorinated ether sulfonate (6:2 Cl-PFESA). Detection frequencies, limits of detection, and analytical performance for all 22 measured PFAS, including compounds not included in the statistical analyses, are provided in Table S2.

Serum PFAS concentrations were measured using a validated solid-phase extraction (SPE) method coupled with ultra-performance liquid chromatography-tandem mass spectrometry (UPLC-MS/MS), as described previously [17]. Briefly, 100 μL of serum was spiked with isotope-labeled internal standards and incubated overnight. Samples were diluted with 4% phosphoric acid and purified using Poly-Sery PWAX SPE cartridges (150 mg, 6 mL; Anpu Experimental Technology Co., Shanghai, China). Chromatographic separation was performed on a ZORBAX Eclipse Plus C18 column (2.1 mm × 150 mm, 1.8 μm; Agilent Technologies, CA, USA) using water and methanol as mobile phases.

Quality assurance and quality control were performed using procedural blanks, solvent blanks, and standard reference material (Organic Contaminants in Non-fortified Human Serum, SRM 1957, National Institute of Standards and Technology, MD, USA) in each batch of 50 samples. Accuracy and precision were within the certified reference ranges provided by the SRM manufacturer.

### 2.4. Statistical Analysis

PFASs with detection rates above 75% were included in the statistical analyses. Concentrations below the limit of detection (LOD) were replaced with LOD/2. Because serum PFAS concentrations showed non-normal distributions according to the Kolmogorov–Smirnov test, natural logarithmic transformation was applied before regression analyses.

Continuous variables were compared using the Mann–Whitney U test, and categorical variables were compared using the chi-square test. Pearson correlation coefficients were calculated to examine correlations among individual PFAS compounds. Multivariable linear regression models were used to evaluate associations between dietary intake variables, sociodemographic characteristics, lifestyle factors, and serum PFAS concentrations. Selection of potential confounders was informed by a directed acyclic graph constructed based on prior knowledge and the study design (Figure S1). In the DAG, dietary intake was specified as the exposure, and serum PFAS concentration was specified as the outcome. The main covariate nodes included population characteristics and related sociodemographic, lifestyle, and physiological factors, including age, ethnicity, education level, annual household income, marital status, fertility status, BMI, smoking, and alcohol consumption. These variables were assumed to be potential common causes of dietary intake patterns and serum PFAS concentrations because they may influence food choices, seafood accessibility, lifestyle-related exposure pathways, PFAS distribution, and PFAS elimination. Based on this DAG, the final adjustment set was selected to reduce confounding while avoiding adjustment for variables that could plausibly lie downstream of dietary intake. Models were adjusted for age, BMI, education level, annual household income, marital status, fertility status, smoking, and alcohol consumption. Additional lifestyle factors, including physical activity and sleep-related variables, were further included as appropriate in sensitivity or fully adjusted models.

To examine whether dietary associations differed by sex, sex-stratified regression analyses were further conducted. To formally assess sex-related effect modification, interaction terms between binary dietary intake variables and sex were added to the fully adjusted multivariable regression models, and *p*-values for dietary intake × sex interaction were calculated. Sex-stratified analyses were used to describe association patterns within males and females, whereas interaction tests were used to evaluate whether these associations differed statistically by sex. Regression results were presented as exponentiated coefficients [exp(β)] with 95% confidence intervals. Because serum PFAS concentrations were natural log-transformed before regression analyses, exp(β) can be interpreted as the adjusted geometric mean ratio of serum PFAS concentrations. Percentage changes were calculated as [exp(β) − 1] × 100%. All statistical analyses were performed using EmpowerStats (X&Y Solutions Inc., Boston, MA, USA) and R software version 4.4.1. A two-tailed *p*-value < 0.05 was considered statistically significant.

## 3. Results

### 3.1. Sex-Linked Characteristics Relevant to PFAS Body Burden

Demographic and lifestyle characteristics of the 1292 participants (493 males, 799 females) are summarized by sex in Table 1. Sex-related differences were particularly evident for variables potentially relevant to PFAS exposure or body-burden variation, including age, BMI, smoking, and alcohol consumption.

Females were younger on average than males, with a higher proportion classified as youth (<35 years; 46.2% versus 31.0%), whereas males had a higher proportion of middle-aged and elderly participants. BMI distribution also differed markedly by sex. Females were more likely to have a normal BMI (18.5–23.9 kg/m^2^; 75.0% versus 46.2%), whereas overweight or obesity (BMI > 23.9 kg/m^2^) was more common among males (51.9% versus 15.5% in females; *p* < 0.001). Because BMI may be related to PFAS distribution and toxicokinetic behavior, this difference provides relevant background for subsequent adjusted analyses.

Lifestyle behaviors showed clear sex-related differences. Smoking prevalence was substantially higher in males than in females (43.6% versus 20.9%; *p* < 0.001), and alcohol consumption showed a similar pattern (52.7% versus 13.3%; *p* < 0.001). These differences are relevant because smoking and alcohol use may reflect lifestyle-related exposure pathways or physiological factors associated with PFAS body burden. Education level also differed by sex, whereas marital status, fertility status, and annual household income were broadly comparable between males and females.

Overall, the observed differences in age, BMI, smoking, and alcohol consumption provide important background for interpreting sex-related differences in serum PFAS concentrations and together support the subsequent sex-stratified analyses.

### 3.2. Dietary Intake Patterns and Serum PFAS Concentrations

**Figure 1:**
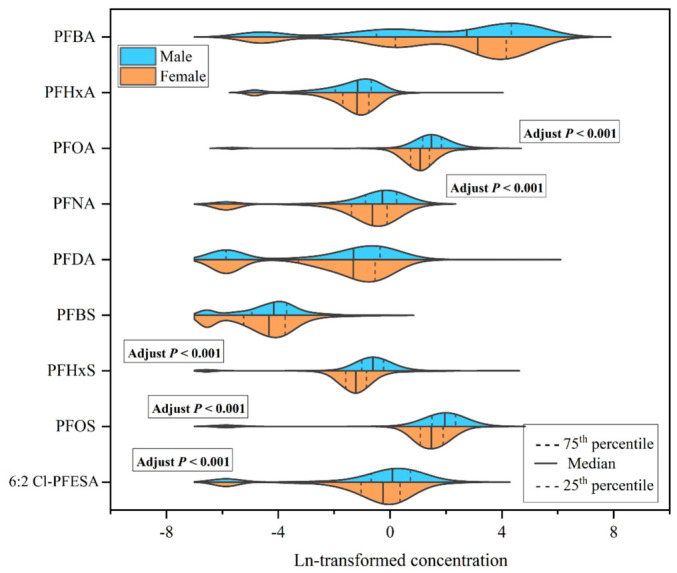
Distribution of serum concentrations of nine PFAS in the study population, stratified by sex. PFAS concentrations were natural log-transformed before visualization. Sex differences were assessed using the Mann–Whitney U test. PFBA, perfluorobutanoic acid; PFHxA, perfluorohexanoic acid; PFOA, perfluorooctanoic acid; PFNA, perfluorononanoic acid; PFDA, perfluorodecanoic acid; PFBS, perfluorobutane sulfonate; PFHxS, perfluorohexanesulfonic acid; PFOS, perfluorooctane sulfonate; 6:2 Cl-PFESA, 9-chlorohexadecafluoro-3-oxanonane-1-sulfonate.

Serum PFAS concentrations varied according to dietary intake frequency across several food groups (Figure 1). Participants reporting more frequent consumption of seafood-related foods, particularly shellfish, shrimp, crabs, fish, and seaweed, generally exhibited higher concentrations of multiple PFAS compounds. Positive associations were especially apparent for PFDA, PFNA, PFHxS, PFOS, and PFOA.

The overall serum PFAS profile provided background support for these dietary associations. As shown in Table S1, most target PFASs were frequently detected in the study population, indicating widespread background exposure, while several long-chain PFASs showed evident population variability. Correlation analysis further showed positive correlations among multiple PFAS compounds after logarithmic transformation (Table S3). The strongest correlations were observed between PFOS and 6:2 Cl-PFESA (r = 0.584), PFOA and PFHxS (r = 0.569), PFHxS and PFOS (r = 0.556), and PFOA and PFOS (r = 0.500). Moderate positive correlations were also observed among several long-chain PFASs, including PFOA–PFNA, PFNA–PFDA, and PFDA–6:2 Cl-PFESA. These correlation patterns may reflect shared exposure sources, co-occurring PFAS mixture profiles, or similar persistence and serum-retention characteristics among long-chain PFAS and selected emerging substitutes. In contrast, correlations involving short-chain PFAS, particularly PFBA and PFBS, were generally weaker, suggesting potentially different exposure sources or toxicokinetic behavior.

Aquatic food consumption showed the most consistent relationships with PFAS concentrations among all evaluated food groups (Figure 1). Shellfish and crustacean intake appeared to exhibit the strongest associations, suggesting that these aquatic food categories may be associated with a disproportionate share of internal PFAS burden. By contrast, vegetables, tea, and processed foods demonstrated comparatively weaker and less consistent associations with serum PFAS concentrations.

Distinct sex-related differences in PFAS distribution patterns were also observed (Figure 1). Males generally exhibited higher concentrations of several long-chain PFAS compounds, including PFOS, PFOA, PFNA, PFDA, and PFHxS, compared with females. Similar patterns were observed for the emerging PFAS compound 6:2 Cl-PFESA. These findings suggest that seafood-related dietary patterns may be associated with PFAS body burden in inland Chinese populations and support subsequent analyses examining whether these food-related associations differed between males and females.

### 3.3. Multivariable Associations of Dietary, Demographic, and Lifestyle Factors with PFAS Body Burden

**Figure 2:**
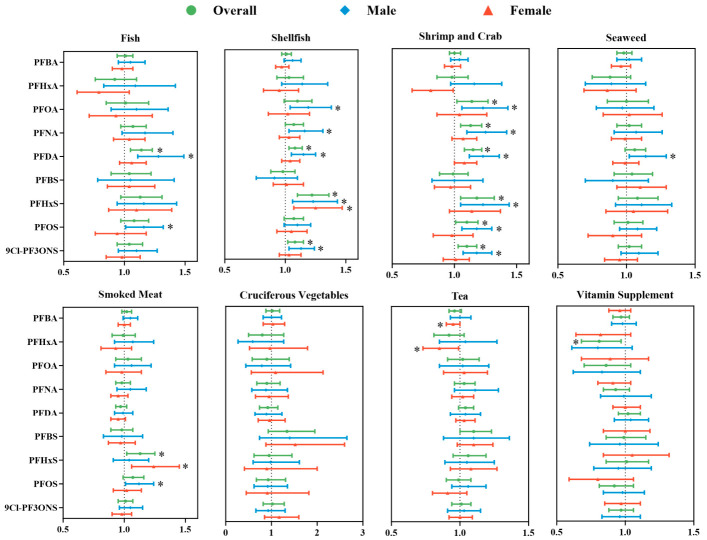
Sex-stratified associations between dietary intake frequency and serum PFAS concentrations. Associations were estimated using multivariable linear regression models adjusted for age, BMI, smoking, alcohol consumption, education level, marital status, fertility status, and annual household income. Dietary intake frequency was originally classified into four categories: never, <1 time per week, 1–2 times per week, and ≥3 times per week. For regression analyses, dietary variables were dichotomized as high-frequency intake (≥1 time per week) and low-frequency intake (<1 time per week). * *p* < 0.05 indicates statistical significance of the association within the overall, male, or female model. Formal sex differences in dietary associations were evaluated using dietary intake × sex interaction terms in fully adjusted models, with *p* values presented in Table S4.

After adjustment for age, BMI, education, income, marital status, smoking, alcohol consumption, physical activity, and sleep-related factors, seafood-related food groups remained consistently associated with elevated serum PFAS concentrations (Figure 2). The main pattern was a positive association between aquatic-food intake and long-chain PFAS, particularly PFDA, PFNA, PFHxS, PFOS, and PFOA. Among the seafood-related categories, shellfish and shrimp/crab intake showed the most consistent and relatively stronger associations, whereas fish and seaweed intake showed similar but comparatively weaker positive patterns. Shrimp and crab intake was also positively associated with the emerging PFAS substitute 6:2 Cl-PFESA, suggesting that crustacean consumption may be relevant not only to legacy long-chain PFAS but also to selected emerging PFAS compounds.

Several demographic and lifestyle factors were also associated with PFAS body burden (Table 2). Older participants exhibited significantly higher concentrations of PFNA, PFDA, and PFHxS compared with younger participants. Higher BMI was positively associated with PFHxS concentrations, particularly among overweight or obese individuals (BMI > 23.9). Alcohol consumption was positively associated with PFOA, PFNA, PFHxS, and PFOS concentrations. In addition, higher household income was associated with elevated PFDA concentrations, whereas marital and fertility status showed compound-specific associations with PFNA, PFDA, PFOA, and PFOS.

Overall, the multivariable regression results highlight seafood-related intake, especially shellfish and shrimp/crab consumption, as the most consistent dietary correlate of elevated serum PFAS concentrations. The direction and relative magnitude of these associations were most evident for long-chain PFASs and, for shrimp/crab intake, also extended to 6:2 Cl-PFESA. These findings provide the main basis for the subsequent sex-stratified analyses.

### 3.4. Sex-Stratified Associations Between Dietary Intake and PFAS Concentrations with Interaction Testing

Associations between seafood-related food groups and PFAS concentrations differed between males and females (Figure 2). Positive associations were particularly evident for shellfish and crustacean intake in relation to PFDA, PFNA, PFOA, PFHxS, and PFOS among males. In females, associations between seafood intake and PFAS concentrations were generally weaker and less consistent.

To formally evaluate whether these associations differed by sex, dietary intake frequency × sex interaction terms were further included in the fully adjusted models (Table S4). Significant interactions were observed for selected diet–PFAS combinations. Among aquatic-food-related variables, shellfish intake showed a significant interaction with sex for PFHxA; shrimp and crab intake showed significant interactions with sex for PFHxA, PFDA, PFOS, and 6:2 Cl-PFESA; and seaweed intake showed significant interactions with sex for PFDA and PFOS. Fish intake did not show statistically significant interactions with sex for the nine PFAS after dichotomization of intake frequency. For non-aquatic dietary variables, significant interactions were observed only for selected combinations, including cruciferous vegetables with PFHxS, tea with PFBS, and vitamin supplement use with PFBA.

These results indicate that sex-related heterogeneity was present for some, but not all, diet–PFAS associations, with more consistent interaction signals observed for selected aquatic-food-related variables. However, these sex-stratified and interaction findings should be interpreted cautiously. Observed differences may reflect not only biological mechanisms related to PFAS toxicokinetics, but also unmeasured behavioral and environmental factors, such as seafood portion size, specific aquatic food species consumed, food sourcing, occupational activities, and consumer product use.

## 4. Discussion

In this cross-sectional study of adults from Sichuan Province, we observed associations between dietary intake, lifestyle characteristics, and serum PFAS concentrations. The most consistent finding was that aquatic-food-related intake, particularly shellfish and shrimp/crab consumption, was positively associated with several long-chain PFAS, including PFDA, PFNA, PFHxS, PFOS, and PFOA. Shrimp/crab intake was also associated with higher concentrations of 6:2 Cl-PFESA. Sex-stratified analyses suggested that some of these associations were more apparent among males, and formal interaction testing provided evidence of sex-related heterogeneity for selected diet–PFAS combinations. In addition, demographic and lifestyle factors, including age, BMI, alcohol consumption, income, marital status, and fertility status, were associated with variations in PFAS body burden. Together, these findings suggest that dietary and lifestyle characteristics may be associated with differences in PFAS body-burden profiles in the present inland Sichuan study population.

Dietary factors are increasingly reported to be associated with PFAS body burden in the general population. Our findings further suggest that aquatic food consumption is associated with internal PFAS levels in this inland population. Frequent consumption of shellfish, shrimp, crabs, fish, and seaweed was associated with higher concentrations of several long-chain PFAS compounds. Among the measured compounds, PFOS, PFOA, PFNA, and PFDA showed particularly consistent associations with seafood-related dietary patterns, consistent with previous biomonitoring studies identifying aquatic food consumption as being associated with long-chain PFAS concentrations [13,18,19]. Shellfish and crustaceans exhibited the strongest associations with PFAS concentrations, suggesting that specific aquatic food categories may be more strongly associated with PFAS body burden than other evaluated food groups. However, because PFAS concentrations were not measured in the consumed food items, these dietary variables should be interpreted as intake-related markers associated with serum PFAS concentrations rather than direct evidence that aquatic foods were the contamination source. This interpretation is consistent with previous evidence that PFASs can occur in aquatic food webs and be detected in aquatic organisms consumed by humans.

Positive associations between aquatic-food-related intake and 6:2 Cl-PFESA are noteworthy because this emerging PFAS substitute has been increasingly detected in Chinese aquatic environments and food products [20,21,22]. Importantly, our findings provide dataset-specific evidence from adults in an inland Chinese province, suggesting that seafood accessibility and dietary diversification may be related to distinct PFAS body-burden profiles associated with aquatic food consumption even outside coastal settings. However, this interpretation should be regarded as hypothesis-generating rather than as definitive evidence of a dietary transition effect in inland populations more broadly. By contrast, vegetables, tea, and processed foods showed comparatively weaker associations with PFAS concentrations in this population. This may reflect differences in dietary patterns, exposure contexts, or unmeasured food-contaminant profiles relative to aquatic foods.

Associations between seafood-related dietary patterns and PFAS concentrations differed between males and females. Positive associations were generally more pronounced among males, particularly for PFDA, PFNA, PFOA, PFHxS, and PFOS. Formal dietary intake × sex interaction analyses further indicated that some associations differed statistically by sex, especially for selected shellfish, shrimp/crab, and seaweed intake variables. One possible explanation is that males may consume larger quantities of seafood and animal-source foods than females, as reported in previous dietary surveys [23]. Physiological differences may also contribute to these patterns. Females may partially eliminate PFAS through menstruation, pregnancy, and breastfeeding, whereas males lack these elimination pathways, potentially resulting in slower PFAS clearance and higher measured body burdens [24,25]. However, the sex-specific patterns should be interpreted cautiously. Observed differences may reflect not only biological mechanisms, but also unmeasured behavioral and environmental factors, including seafood portion size, specific aquatic food species consumed, food sourcing, occupational activities, household environmental conditions, and consumer product use. Therefore, the sex-stratified findings should not be interpreted as evidence of purely biological sex-specific effects, but rather as potential effect modification that warrants confirmation in future studies.

Several demographic and lifestyle factors were also associated with PFAS concentrations. Older participants exhibited higher concentrations of PFNA, PFDA, and PFHxS, which may reflect cumulative exposure and the relatively long biological half-lives of these compounds. Notably, higher BMI was positively associated with PFHxS concentrations. Previous studies have suggested that PFHxS may exhibit distinct toxicokinetic behavior compared with other PFAS compounds because of its relatively long biological half-life and potential interactions with lipid and metabolic pathways. Individuals with overweight or obesity may have different PFAS distribution patterns. Positive associations between alcohol consumption and PFAS levels were observed for PFOA, PFNA, PFHxS, and PFOS. Similar findings have been reported in NHANES analyses and other epidemiological studies [26]. Alcohol consumption may be associated with hepatic stress and altered metabolic processing, which could in turn be related to PFAS elimination efficiency (though causal inference is not possible from cross-sectional data) [27,28]. Associations with marital and fertility status were also observed for several PFAS compounds, although the underlying mechanisms remain unclear and may partly reflect differences in age structure, household dietary patterns, and reproductive-related PFAS elimination.

Our findings highlight a potential food-safety and public health concern that warrants further investigation through longitudinal dietary assessment and direct PFAS monitoring in foods. Aquatic foods provide high-quality protein, essential fatty acids, and important micronutrients, and are widely promoted as components of healthy dietary patterns. However, PFASs may be present in some aquatic foods, raising food-contaminant concerns that should be considered alongside their nutritional benefits. The coexistence of nutritional benefits and chemical burdens underscores a potential trade-off within contemporary food systems, particularly in populations undergoing rapid dietary transition and increasing seafood consumption. These findings may further support the value of integrating human biomonitoring with food-contaminant surveillance, particularly for aquatic foods consumed in inland regions. Future contaminant monitoring of aquatic foods would help determine whether the dietary associations observed in this study correspond to measurable PFAS contamination in foods. Risk-benefit communication strategies may also be needed to support informed dietary choices in populations experiencing rapid dietary diversification.

This study has several strengths. We simultaneously evaluated multiple PFAS compounds, dietary factors, demographic characteristics, and lifestyle variables in a relatively large population from an inland Chinese province. Sex-stratified analyses, together with dietary intake × sex interaction testing, allowed assessment of potential sex-related heterogeneity in diet–PFAS associations. In addition, multivariable regression models were used to control for multiple confounders, improving the robustness of the findings.

Several limitations should also be considered. The cross-sectional design limits causal inference and prevents assessment of temporal relationships between dietary intake and PFAS body burden. The study population was recruited from a hospital setting in a single inland province, which may introduce selection bias and limit extrapolation to other regions, populations, and dietary contexts. Therefore, the findings should be interpreted as reflecting a specific regional dietary transition context in Sichuan rather than broader national or global dietary patterns.

Dietary intake frequency was self-reported and may therefore be subject to recall bias or exposure misclassification. In addition, dietary intake frequency was dichotomized as high-frequency intake (≥1 time per week) and low-frequency intake (<1 time per week) for regression analyses. While this approach improved model stability and distinguished regular from non-regular intake, it inevitably reduced exposure granularity and limited the ability to capture potential dose–response relationships. Beyond measurement-related limitations, residual confounding from unmeasured environmental and behavioral factors cannot be excluded. Water source may be an important confounder because PFAS concentrations can vary across municipal water, groundwater, bottled water, and locally contaminated water supplies [29]. If individuals with higher aquatic-food intake also differed in their drinking-water sources or residential environments, part of the observed diet–PFAS associations may reflect shared environmental exposure rather than dietary intake alone. Similarly, occupational exposure related to fluorochemical production, textile processing, metal plating, firefighting materials, or other PFAS-related industries may contribute to background exposure variability. Local environmental contamination driven by industrial activity, wastewater discharge, and hydrological conditions may further influence both food contamination and human exposure [30]. Because these factors were not directly measured in the present study, these factors could not be fully controlled. Future studies incorporating water-source information, occupational history, geospatial environmental data, and environmental monitoring would therefore help to better disentangle dietary and non-dietary exposure pathways.

An additional consideration relates to exposure characterization. PFAS concentrations were not measured in the foods consumed by participants; therefore, aquatic-food intake should be interpreted as an intake-related indicator associated with serum PFAS concentrations rather than direct evidence of contaminated food sources. Moreover, the dietary questionnaire captured only broad aquatic-food categories and did not include information on seafood species, geographic origin, production systems, sourcing information, portion size, or cooking methods. This may have introduced exposure misclassification, as PFAS levels can vary substantially across species and production environments, leading to heterogeneity in actual exposure among participants with similar reported intake frequencies.

Finally, multiple association and interaction tests were performed across dietary variables and PFAS compounds, which may increase the likelihood of chance findings. No formal multiple-comparison correction was applied because the analyses were exploratory and hypothesis-generating in nature. Therefore, interpretation of statistical significance should rely on the consistency of observed patterns across related food groups and PFAS compounds rather than isolated *p*-values. Accordingly, sex-related interaction findings in particular should be interpreted with caution and require confirmation in future studies.

Future research integrating detailed dietary assessment, species-level exposure data, food-contaminant measurements, water-source information, occupational history, environmental surveillance, and repeated biomarker sampling will be essential to better characterize dietary and non-dietary contributions to PFAS body burden. From a risk-reduction perspective, the present findings may further support continued monitoring of PFAS in aquatic food supply chains rather than immediate changes in seafood consumption behavior. Because PFAS profiles may vary substantially across species and production environments, future surveillance efforts could prioritize frequently consumed aquatic foods, particularly shellfish and crustaceans. Integrating contaminant monitoring with dietary assessment and nutritional considerations will be important for developing balanced risk-communication strategies.

## 5. Conclusions

In this cross-sectional study, seafood consumption was associated with higher serum PFAS concentrations, with stronger associations generally observed among males. These findings should be interpreted as associations rather than evidence of causal or temporal relationships between seafood intake and PFAS body burden. The observed patterns suggest that seafood-related dietary patterns may be linked to differences in PFAS body-burden profiles in this inland Sichuan study population, where dietary diversification and increased access to aquatic foods may be occurring.

Given the nutritional importance of aquatic foods, our findings highlight a potential trade-off between dietary benefits and food-contaminant concerns. However, the quantitative findings should not be directly extrapolated to broader national or global populations. Rather, the observed association patterns provide hypothesis-generating evidence for similar inland regions experiencing dietary diversification and increased access to aquatic foods. These findings may provide additional evidence supporting continued PFAS monitoring in aquatic food supply chains, particularly species-specific surveillance combined with dietary exposure assessment, while helping to inform balanced risk-reduction strategies without undermining the nutritional benefits of seafood consumption. Future longitudinal and multi-region studies incorporating detailed dietary assessment, direct PFAS measurements in consumed foods, and environmental monitoring are needed to clarify whether and how dietary diversification contributes to PFAS body-burden patterns over time.

## Figures and Tables

**Table 1:** Demographic and lifestyle characteristics of the study participants (n = 1292).

Characteristic	Total Sample	Male (n = 493)	Female (n = 799)	*p* Value
**Age (years)**				<0.001
Youth (<35)	522 (40.4%)	153 (31.0%)	369 (46.2%)	
Middle-aged (35–50)	568 (44.0%)	241 (48.9%)	327 (40.9%)	
Elderly (>50)	202 (15.6%)	99 (20.1%)	103 (12.9%)	
**Body mass index (kg/m^2^)**				<0.001
<18.5	85 (6.6%)	9 (1.8%)	76 (9.5%)	
18.5–23.9	827 (64.0%)	228 (46.2%)	599 (75.0%)	
>23.9	380 (29.4%)	256 (51.9%)	124 (15.5%)	
**Education level**				<0.001
College and below	942 (72.9%)	293 (59.4%)	649 (81.2%)	
College above	350 (27.1%)	200 (40.6%)	150 (18.8%)	
**Marital status**				
Spinsterhood	230 (17.8%)	92 (18.7%)	138 (17.3%)	
Married	1062 (82.2%)	401 (81.3%)	661 (82.7%)	
**Fertility status**				
Childlessness	267 (20.6%)	101 (20.5%)	166 (20.8%)	
One child	780 (60.4%)	290 (58.8%)	490 (61.3%)	
Two children	245 (19.0%)	102 (20.7%)	143 (17.9%)	
**Annual household income**				
<200,000	814 (63.0%)	307 (62.3%)	507 (63.5%)	
200,000–300,000	281 (21.7%)	103 (20.9%)	178 (22.3%)	
>300,000	197 (15.2%)	83 (16.8%)	114 (14.3%)	
**Whether smoking**				<0.001
Yes	382 (29.6%)	215 (43.6%)	167 (20.9%)	
No	910 (70.4%)	278 (56.4%)	632 (79.1%)	
**Whether drinking**				<0.001
Yes	366 (28.3%)	260 (52.7%)	106 (13.3%)	
No	926 (71.7%)	233 (47.3%)	693 (86.7%)	

Data are presented as numbers (percentages) of participants. *p*-values indicate differences between men and women and were calculated using the chi-square test for categorical variables. BMI was calculated by dividing weight (kg) by the square of height (m^2^). The annual household income was measured in CNY per year.

**Table 2:** Multivariable associations of demographic and lifestyle factors with serum PFAS concentrations.

	PFBA	PFHxA	PFOA	PFNA	PFDA	PFBS	PFHxS	PFOS	6:2 Cl-PFESA
**Age**									
Middle-aged	0.98 (0.94, 1.02)	0.91 (0.81, 1.02)	0.89 (0.76, 1.03)	1.05 (0.97, 1.14)	0.99 (0.94, 1.05)	0.93 (0.84, 1.03)	1.21 (1.04, 1.34) *	1.01 (0.91, 1.13)	1.08 (1.00, 1.18)
Elderly	0.98 (0.92, 1.03)	0.78 (0.67, 0.91) **	0.83 (0.67, 1.02)	1.19 (1.04, 1.36) **	1.10 (1.01, 1.20) *	1.03 (0.90, 1.18)	1.38 (1.13, 1.70) **	1.03 (0.88, 1.19)	1.02 (0.90, 1.15)
**BMI**									
18.5–23.9	0.98 (0.91, 1.07)	0.80 (0.63, 1.03)	0.97 (0.76, 1.24)	1.08 (0.94, 1.24)	1.01 (0.90, 1.13)	0.84 (0.69, 1.01)	1.21 (0.95, 1.54)	0.92 (0.75, 1.14)	1.09 (0.95, 1.25)
>23.9	0.98 (0.90, 1.06)	0.65 (0.50, 0.84) **	1.15 (0.87, 1.53)	1.09 (0.94, 1.27)	0.96 (0.85, 1.08)	0.92 (0.76, 1.12)	1.55 (1.19, 2.02) **	0.861 (0.69, 1.08)	1.11 (0.95, 1.29)
Education level	1.02 (0.98, 1.06)	0.98 (0.88, 1.09)	0.94 (0.85, 1.04)	0.94 (0.88, 1.00)	0.99 (0.94, 1.04)	0.90 (0.82, 1.00) *	0.96 (0.87, 1.06)	0.99 (0.92, 1.07)	1.01 (0.95, 1.07)
Marital status	1.02 (0.97, 1.06)	1.01 (0.90, 1.14)	0.95 (0.83, 1.09)	1.19 (1.11, 1.27) **	1.17 (1.11, 1.25) **	0.96 (0.86, 1.08)	1.01 (0.89, 1.14)	1.09 (1.01, 1.18) *	1.09 (1.02, 1.17) *
**Fertility status**									
One child	1.01 (0.96, 1.06)	1.02 (0.89, 1.16)	0.71 (0.59, 0.85) **	1.19 (1.09, 1.30) **	1.08 (1.01, 1.16) *	0.88 (0.78, 0.99) *	1.09 (0.92, 1.28)	1.12 (0.99, 1.27)	1.03 (0.93, 1.13)
Two children	1.05 (0.99, 1.11)	0.98 (0.83, 1.15)	0.84 (0.69, 1.03)	1.10 (0.98, 1.22)	1.18 (1.08, 1.29) **	0.92 (0.80, 1.07)	0.91 (0.76, 1.10)	1.05 (0.91, 1.21)	0.99 (0.88, 1.11)
**Annual household income**									
200,000–300,000	0.99 (0.95, 1.04)	1.01 (0.89, 1.15)	1.07 (0.90, 1.26)	0.98 (0.90, 1.08)	1.07 (1.00, 1.15) *	0.95 (0.85, 1.07)	1.05 (0.90, 1.24)	0.97 (0.86, 1.10)	1.03 (0.94, 1.14)
>300,000	1.00 (0.94, 1.05)	0.93 (0.80, 1.07)	0.98 (0.81, 1.20)	0.94 (0.85, 1.04)	1.17 (1.08, 1.28) **	0.97 (0.85, 1.11)	1.18 (0.98, 1.43)	1.07 (0.91, 1.25)	0.96 (0.86, 1.07)
**Whether smoking**	0.99 (0.95, 1.02)	0.90 (0.81, 0.99) *	1.00 (0.90, 1.11)	1.05 (0.98, 1.12)	0.99 (0.94, 1.04)	1.06 (0.97, 1.17)	1.08 (0.97, 1.21)	1.05 (0.97, 1.14)	1.01 (0.95, 1.07)
**Whether drinking**	1.00 (0.96, 1.04)	0.98 (0.88, 1.09)	1.24 (1.08, 1.41) **	1.12 (1.04, 1.20) **	1.01 (0.96, 1.06)	1.08 (0.98, 1.20)	1.37 (1.20, 1.57) **	1.10 (1.01, 1.20) *	1.04 (0.98, 1.11)

The dependent variable was the natural log-transformed serum PFAS concentration. Values are presented as exponentiated regression coefficients [exp(β)] with 95% confidence intervals and can be interpreted as adjusted geometric mean ratios. Percentage changes can be calculated as [exp(β) − 1] × 100%. Reference categories were youth, BMI <18.5 kg/m^2^, childlessness, and annual household income <200,000 CNY, * *p* < 0.05, ** *p* < 0.01.

## Data Availability

The data supporting the findings of this study are included in the article and Supplementary Materials. Additional data are available from the corresponding authors upon reasonable request, subject to ethical and privacy restrictions.

## Supplementary Materials

The following supporting information can be downloaded at: https://www.mdpi.com/article/10.3390/foods15142472/s1, Figure S1: The directed acyclic graph (DAG) for all potential confounders considered in the statistical analyses; Table S1: Detection rate and concentration of PFASs in the study population (n = 1292, ng/mL); Table S2: Detection frequency, limits of detection, and analytical performance of the 22 PFASs measured in serum; Table S3: Pearson correlation coefficients between PFAS concentrations in human serum samples after logarithmic transformation in this study; Table S4: Dietary intake frequency × sex interaction analysis for serum PFAS concentrations.
