# Supplementary material for: Seafood-Linked and Sex-Specific Signatures of Legacy and Emerging PFAS Body Burden During Dietary Transition in Sichuan, China
Source: Foods. 2026 Jul 13;15(14):2472. doi: 10.3390/foods15142472 (PMC13409660; doi:10.3390/foods15142472)
Supplement: Supplementary file 1 [file foods-15-02472-s001.zip › foods-4407209-supplementary.pdf]

Table S1. Detection rate and concentration of PFASs in the study population (n = 1292, ng/mL).

PFASs	DR(%)	Percentiles				GM
		P25	P50	P75	P95	
PFBA	89.01	1.0175	21.2330	66.9500	208.0513	6.1908
PFHxA	94.97	0.1658	0.3089	0.4869	0.8151	0.2085
PFOA	98.22	2.3371	3.4791	5.0573	10.5347	3.3139
PFNA	89.94	0.3042	0.6124	1.0218	2.0375	0.3036
PFDA	75.23	0.0028	0.2705	0.6184	1.3678	0.2099
PFBS	88.85	0.0059	0.0139	0.0242	0.0731	0.0265
PFHxS	97.60	0.2388	0.3628	0.5757	1.3335	0.3598
PFOS	96.44	3.3519	5.2405	8.0870	14.4594	4.6647
9Cl-PF3ONS	91.02	0.4002	0.8921	1.6416	3.9210	1.5126

Note:DR, Detection Rate; GM,Geometric Mean.

Table S2. Detection frequency, limits of detection, and analytical performance of the 22 PFASs measured in serum.

PFAS	LOD (ng/mL)	R^2	Recovery (%)	DR(%)
PFBA	0.014	0.9990	78	89.01
PFPeA	0.013	0.9977	116	59.67
PFHxA	0.011	0.9973	106	94.97
PFHpA	0.007	0.9990	98	43.19
PFOA	0.005	0.9996	103	98.22
PFNA	0.004	0.9969	114	89.94
PFDA	0.004	0.9978	70	75.23
PFUdA	0.004	0.9969	71	34.91
PFDoA	0.005	0.9979	117	4.02
PFTrDA	0.003	0.9961	96	5.26
PFTeDA	0.005	0.9977	111	26.70
PFBS	0.002	0.9994	91	88.85
PFHxS	0.002	0.9976	61	97.60
PFOS	0.004	0.9963	74	96.44
PFDS	0.007	0.9991	68	40.33
NaDONA	0.001	0.9994	65	3.64
6:2 Cl-PFESA	0.001	0.9966	73	91.02
8:2 Cl-PFESA	0.001	0.9977	78	4.18
N-EtFOSAA	0.006	0.9990	118	6.58
4:2 FTS	0.033	0.9978	71	51.24
6:2 FTS	0.006	0.9987	71	32.51
8:2 FTS	0.004	0.9973	66	1.63

Note: LOD, limit of detection; R^2, coefficient of determination. The 22 PFAS showed good linearity over the tested calibration range, with R^2 values of 0.9961 – 0.9996. 6:2 Cl-PFESA is also referred to as 9Cl-PF3ONS.

Table S3. Pearson correlation coefficients between PFAS concentrations in human serum samples after logarithmic transformation in this study.

PFASs	PFBA	PFHxA	PFOA	PFNA	PFDA	PFBS	PFHxS	PFOS	9Cl-PF3ONS
PFBA	1								
PFHxA	0.159*	1							
PFOA	0.126*	0.327*	1						
PFNA	-0.074*	0.099*	0.437*	1					
PFDA	0.054	0.093*	0.296*	0.384*	1				
PFBS	0.012	0.034	0.188*	0.211*	0.058	1			
PFHxS	0.154*	0.275*	0.569*	0.238*	0.247*	0.086*	1		
PFOS	0.058	0.323*	0.500*	0.350*	0.326*	0.093*	0.556*	1	
9Cl-PF3ONS	0.016	0.181*	0.359*	0.419*	0.462*	0.013	0.386*	0.584*	1

Note: Statistically significant (P-value < 0.001).

Table S4. Dietary intake frequency × sex interaction analysis for serum PFAS concentrations.

PFAS	Fish	Shellfish	Shrimp and crab	Seaweed	Smoked meat	cruciferous vegetables	tea	vitamin supplement
PFBA	0.066	0.131	0.524	0.445	0.394	0.902	0.812	0.026*
PFHxA	0.349	0.047*	0.001*	0.398	0.594	0.319	0.430	0.478
PFOA	0.503	0.071	0.119	0.831	0.245	0.210	0.231	0.784
PFNA	0.651	0.107	0.362	0.522	0.663	0.987	0.830	0.670
PFDA	0.737	0.003*	0.004*	0.007*	0.482	0.246	0.458	0.452
PFBS	0.082	0.767	0.552	0.235	0.772	0.776	0.028*	0.595
PFHxS	0.752	0.561	0.394	0.530	0.499	0.020*	0.293	0.925
PFOS	0.139	0.070	0.003*	0.019*	0.828	0.843	0.446	0.854
9Cl-PF3ONS	0.604	0.060	0.018*	0.505	0.239	0.301	0.592	0.515

Note: Values are P values for the dietary intake frequency × sex interaction term. For regression analyses, dietary intake frequency was dichotomized as high-frequency intake (≥1 time per week) and low-frequency intake (<1 time per week), consistent with Figure 2. The dependent variables were natural log-transformed serum PFAS concentrations. P values were obtained from fully adjusted multivariable models including the dietary variable, sex, and the dietary variable × sex interaction term. Models were adjusted for age group, BMI group, education level, annual household income, marital status, fertility status, smoking, alcohol consumption, physical activity, and sleep quality, as applicable. *P for interaction < 0.05.

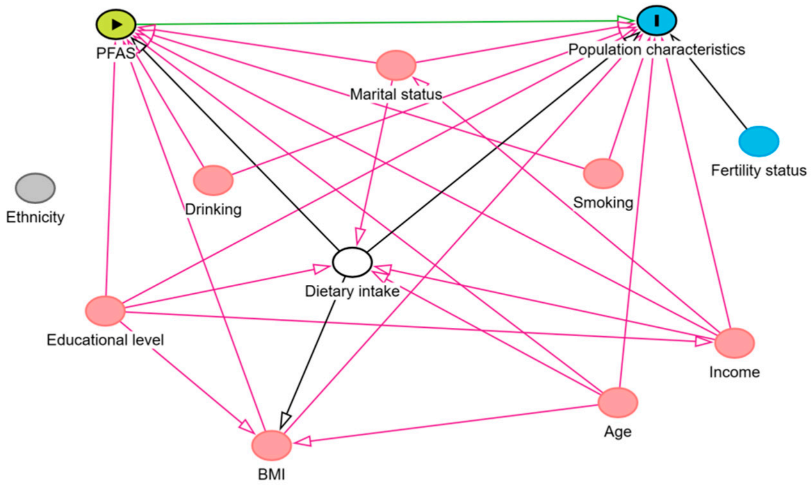

Figure S1. The directed acyclic graph (DAG) for all potential confounders considered in the statistical analyses. Dietary intake was specified as the exposure, and serum PFAS concentration was specified as the outcome. The main covariate nodes included population characteristics and related sociodemographic, lifestyle, and physiological factors, including age, ethnicity, education level, annual household income, marital status, fertility status, BMI, smoking, and alcohol consumption. These variables were considered potential common causes of dietary intake patterns and serum PFAS concentrations because they may influence food choices, seafood accessibility, lifestyle-related exposure pathways, PFAS distribution, and PFAS elimination. The DAG was used to identify an adjustment set for the multivariable regression models.
